# Predicting Cytotoxicity of Metal Oxide Nanoparticles Using Isalos Analytics Platform

**DOI:** 10.3390/nano10102017

**Published:** 2020-10-13

**Authors:** Anastasios G. Papadiamantis, Jaak Jänes, Evangelos Voyiatzis, Lauri Sikk, Jaanus Burk, Peeter Burk, Andreas Tsoumanis, My Kieu Ha, Tae Hyun Yoon, Eugenia Valsami-Jones, Iseult Lynch, Georgia Melagraki, Kaido Tämm, Antreas Afantitis

**Affiliations:** 1 NovaMechanics Ltd., Nicosia 1065, Cyprus; papadiamantis@novamechanics.com (A.G.P.); Voyiatzis@novamechanics.com (E.V.); tsoumanis@novamechanics.com (A.T.); 2 School of Geography, Earth and Environmental Sciences, University of Birmingham, Birmingham B15 2TT, UK; E.ValsamiJones@bham.ac.uk (E.V.-J.); i.lynch@bham.ac.uk (I.L.); 3 Institute of Chemistry, University of Tartu, 50411 Tartu, Estonia; jaak.janes@ut.ee (J.J.); laurisikk@gmail.com (L.S.); jaanus.burk@ut.ee (J.B.); peeter.burk@ut.ee (P.B.); 4 Department of Chemistry, College of Natural Sciences, Hanyang University, Seoul 04763, Korea; hakieumy12@gmail.com (M.K.H.); taeyoon@hanyang.ac.kr (T.H.Y.); 5 Institute of Next Generation Material Design, Hanyang University, Seoul 04763, Korea; 6 Division of Physical Sciences and Applications, Hellenic Military Academy, 16672 Vari, Greece; georgiamelagraki@gmail.com

**Keywords:** cytotoxicity, metal oxide nanoparticles, Isalos analytics platform, computational descriptors, in silico modelling, machine learning, atomistic descriptors

## Abstract

A literature curated dataset containing 24 distinct metal oxide (Me_x_O_y_) nanoparticles (NPs), including 15 physicochemical, structural and assay-related descriptors, was enriched with 62 atomistic computational descriptors and exploited to produce a robust and validated in silico model for prediction of NP cytotoxicity. The model can be used to predict the cytotoxicity (cell viability) of Me_x_O_y_ NPs based on the colorimetric lactate dehydrogenase (LDH) assay and the luminometric adenosine triphosphate (ATP) assay, both of which quantify irreversible cell membrane damage. Out of the 77 total descriptors used, 7 were identified as being significant for induction of cytotoxicity by Me_x_O_y_ NPs. These were NP core size, hydrodynamic size, assay type, exposure dose, the energy of the Me_x_O_y_ conduction band (*E_C_*), the coordination number of the metal atoms on the NP surface (Avg. C.N. Me atoms surface) and the average force vector surface normal component of all metal atoms (v⊥ Me atoms surface). The significance and effect of these descriptors is discussed to demonstrate their direct correlation with cytotoxicity. The produced model has been made publicly available by the Horizon 2020 (H2020) NanoSolveIT project and will be added to the project’s Integrated Approach to Testing and Assessment (IATA).

## 1. Introduction

Naturally occurring nanoscale particles (nanoparticles, NPs) have existed throughout Earth’s and subsequently human history. They can be produced from biological, anthropogenic and geological phenomena, such as erosion, volcanic eruptions and forest fires, charcoal burning, industrial operations and more [1]. In recent years, the increased production of engineered NPs, with tuneable physical, chemical and biological properties, has led to them being widely used, among others, in the automotive, electronics, optics, food technology, cosmetics and healthcare industries [1,2]. Metal oxide (Me_x_O_y_) engineered NPs are among the highest produced per volume and are used widely in technological applications like gas sensors, photovoltaics, adsorbents, catalysis and fuel cells [3,4], due to their unique superparamagnetic, piezoelectric, optical, etc. properties [5,6,7,8,9]. Me_x_O_y_ NPs are also being used in various consumer products, such as food, cosmetics (sunscreen) and electronic and medical devices [10].

Due to their small size and high biochemical activity, and despite their useful and beneficial properties, there are concerns that NPs are able to cross biological barriers and access a wide number of organs and tissues in the human body, including (to a limited extent) the blood–brain barrier [11], which can lead to toxic side-effects [11,12,13,14,15,16,17,18,19]. As a result, the hazard and risk assessment of NPs is key to ensure their safe use for humans and the environment. So far, in vivo studies have been the main source of information regarding NP effects on the physiology of biological organisms [20,21,22]. Furthermore, the requirements for a reduction in animal testing as per the 3Rs (Replace, Reduce, Refine) principles [23] and European Commission (EC) legislation [24] have led to a push for development of alternative testing strategies (ATS). These, so far, have mostly translated to in vitro models, followed by in silico modelling [25,26]. According to a study by Törnqvist et al. (2014), data from 36 relevant projects demonstrated major (i.e., 53%) reductions in the use of animals in pharmaceutical development [27]. Taking into account the cost and working hours associated with in vivo and in vitro experiments and that traditional “wet-lab” toxicology cannot keep up with diversity and increasing abundance of engineered NPs, computational modelling has the potential to act as a high-throughput alternative [28,29] and is becoming increasingly accepted in regulatory testing as model validation and documentation improves.

Several studies have been published assessing chemical and NP toxicity using computational methods [30,31,32,33,34,35,36,37,38,39,40,41,42,43,44,45], the majority of which are based on machine learning techniques and the development of Quantitative Nanostructure-Activity relationships (QNARs) [30,31,36,37,38,41,42,44,45]. The goal of these studies was to use computational methods to understand the mode of action (MOA) of the NPs, and the NP characteristics driving toxicity. Furthermore, it was possible to elucidate the relevant Adverse Outcome Pathways (AOPs), as part of integrated approaches to testing and assessment (IATA). Previous findings have demonstrated that Me_x_O_y_ NPs toxicity can, e.g., originate from dissolution and metal ion “shedding” from the NP shell area [40]. In some cases, the experimental datasets used were complemented with computational descriptors, which were based on atomistic approaches [40,41,45], Simplified Molecular-Input Line-Entry System (SMILES) codes [42,43,44,46,47] and calculation of descriptors using image analysis [48,49]. However, promising, computational studies present certain limitations when it comes to the calculation of atomistic descriptors. Taking into account that NPs with a diameter of 5 nm contain approximately 20,000 atoms, this means that a 30 nm NP will contain over 10^6^ atoms [50]. Therefore, with the current computational power available, calculation of full-particle descriptors for nanoscale particles using ab initio or semiempirical methods is impossible in the near future. Thus, workarounds including approximations and simplified NP models are used [40,41,42,43,51]. Early approaches employed the design of molecular descriptors using SMILES structures and took into account the NPs’ chemical composition and could also include information about the experimental conditions, such as the dispersion medium [42,43,51]. Later studies were based on the quantum-chemical calculation of small atomic clusters [41,52], from which quantum-chemical descriptors such as the HOMO-LUMO gap (also called band gap) and enthalpy of formation, which is currently not possible for full-sized NPs, can be calculated. These descriptors can be directly used to model the toxic properties of NPs without taking into account the size dependency of the descriptors [41], or can be extrapolated to find descriptor values for specific NP sizes [52]. More recent studies [40,53,54] employed molecular mechanics to calculate full descriptors and energetics (such as potential energies and coordination numbers) to model Me_x_O_y_ NPs. Further approaches, implemented NPs surface modification characteristics (e.g., coating polymers and proteins absorption) [55,56,57] to describe more complex systems like competitive protein absorption and NPs exposure to biological systems, thus creating more complete QNAR models.

In this work, we present a meta-analysis of a dataset by Zhang et al. (2012) [45] retrieved from the S^2^NANO (www.s2nano.org) database on the cytotoxicity of 24 Me_x_O_y_ NPs to human bronchial epithelial (BEAS-2B) and murine myeloid (RAW 264.7) cell lines using single parameter (% cell viability) adenosine triphosphate (ATP) and lactate dehydrogenase (LDH) assays. The dataset was enriched with 62 full-particle atomistic descriptors based on the atomic structure of each NP (1488 datapoints), which requires only the crystal structure of the respective bulk material [40,53]. These descriptors were included together with the full dataset in the NanoPharos database (https://db.nanopharos.eu/), developed within the NanoSolveIT [58] and NanoCommons [59] projects. Furthermore, a QNAR model was developed to predict Me_x_O_y_ NP cytotoxicity on these cell lines based on the identified most statistically significant descriptors, providing new insights into the drivers of the Me_x_O_y_ NP toxicity. The fully documented model and guidance documentation on its use has been made publicly available as a webservice (https://cellviability.cloud.nanosolveit.eu/) to ensure accessibility within the scientific community and to interested stakeholders.

## 2. Materials and Methods

### 2.1. Toxicological Data from Metal Oxide NPs

The dataset used for descriptor calculation and model development was retrieved from the S^2^NANO (www.s2nano.org) database. Selection was performed based on the dataset’s NP variability and the quality score [60] assigned by the database curators. The original cytotoxicity experiments, carried out by Zhang et al. (2012) [45], contained 24 different Me_x_O_y_ NPs (17 commercial and 7 synthesised in house) with no data gaps. In addition, physicochemical and structural characterisations (NP core size, specific surface area, total surface area, hydrodynamic size, ζ-potential, point of zero ζ-potential, metal dissolution and crystal structure) were also available for all NPs. The dataset was enriched with molecular descriptors that could be calculated using fundamental atomic parameters. These include the energy of the valence (*E_V_*) and conduction (*E_C_*) bands, the energy band gap (*E_g_*), the metal electronegativity (*χ_cation_*), Me_x_O_y_ absolute electronegativity (*χ_oxide_*) and standard enthalpy of formation (*E_ΔH_*).

The cytotoxicity experiments carried out by Zhang et. al. (2012) [45] demonstrated the possibility to use *E_c_* levels to delineate the toxicological potential of Me_x_O_y_ NPs at the cellular and whole animal level. In vitro toxicological analyses were carried out both in single- and multiparameter toxicity assays. Single-parameter ATP and LDH assays in human bronchial epithelial (BEAS-2B) and murine myeloid (RAW 264.7) cell lines were included as they are commonly used to assess engineered NPs cytotoxicity without reference to a specific MOA [61,62]. Results from these single-parameter toxicity analyses were compared with results from an in-house multiparameter high-throughput screening assay containing 5 parameters measuring oxidative stress [45]. There was strong correlation between the multiparameter and single-parameter responses confirming that the same 7 NPs are potentially more hazardous in general toxicity assays, as well as during comparative analysis of their oxidative stress effects in the multiparameter assays. These findings were further confirmed in an in vivo mouse model, where generation of acute neutrophilic inflammation and cytokine responses in the lungs of C57 BL/6 mice was measured.

In the current study, we used a subset of the data for modelling from the aforementioned single-parameter ATP and LDH assays carried out in BEAS-2B and RAW 264.7 cell lines. The type of assay (ATP or LDH) was included as an extra parameter, since significant correlation was identified in various instances [60,63,64,65] between the type of assay and cytotoxicity results. In total, 15 descriptors (independent variables) originating from Zhang et al. (2012) were included in the analysis: 6 physicochemical (chemical formula, core size, specific surface area, total surface area, hydrodynamic size and ζ-potential), 6 molecular (*E_V_*, *E_C_*, *E_g_*, *χ_cation_*, *χ_oxide_* and *E_ΔH_*) and 3 assay-related (assay type, cell species and NP exposure dose) descriptors. The biological endpoint (dependent variable) was % cell viability 24 h postexposure.

### 2.2. Dataset Enrichment with Computational Descriptors

The dataset resulting from the above analysis was further enriched with 62 computational descriptors for each NP, which are directly related with its stability. We have previously reported a methodology to calculate a set of full particle nanodescriptors, based on the atomic structure of NPs, which requires as input the crystal structure of the respective bulk materials [40,53]. The NP structures were derived from the most thermodynamically stable crystal structures of the respective bulk metal oxides. The unit cells of the metal oxides were replicated in all three dimensions using in-house developed Python scripts and the molecular dynamics software LAMMPS. Full details of the simulation approach are provided in the Modelling Data (MODA) reporting template provided in the electronic Supplementary Information (ESI S2). The resulting spherical NPs were generated by deleting all atoms outside of the set radius of the produced NPs, while performing energy minimisation (using the Lennard-Jones parameters [53]) and maintaining the electroneutrality of the final NPs. Subsequently, the NPs were subjected to energy minimisation using the Polak–Ribiere version of the conjugate gradient (CG) algorithm [66].

Potential energies of atoms were calculated based on the Buckingham [67] and Coulomb potentials using the force field presented in Ref. [68]. Coulombic interactions were calculated using the Wolf summation [68], which is much more computationally affordable than the standard Ewald summation [69]. Cut-off radii for the Wolf summation were derived by matching energies of infinite crystals with small clusters of unit cells (2 × 2 × 2 unit cells). The calculations were performed under periodic boundary conditions in all three cartesian directions employing the LAMMPS software [70]. The length of the simulation box in each direction was much larger than the NP diameter and the cut-off values of the Buckingham potentials and Wolf summation. Thus, it was ensured that there are no interactions of the NP atoms with their periodic images. The derivation of nanodescriptors was based on the core and shell models for spherical NPs. The shell region refers to the atoms located within a depth of 1 nm from the surface of the NP, with the rest forming the NP’s core. This model allows the construction of a number of nanodescriptors that quantify the special features of the surface atoms based on different parameters such as potential energies and coordination numbers.

In total, 62 descriptors were derived from chemical composition (9 descriptors), potential energy (9 descriptors), topology (9 descriptors), lattice energy (5 descriptors), size (3 descriptors) and force vectors (27 descriptors). The atomistic descriptors calculations were performed for the entire atom, the Me_x_O_y_ core and the Me_x_O_y_ shell and can be divided into 3 categories: (1) related to all metal and oxygen atoms contained in the Me_x_O_y_, (2) related to all metal atoms contained into the Me_x_O_y_ and (3) all oxygen atoms contained into the Me_x_O_y_. In all three cases, the computed descriptors included the number of atoms present, the Me_x_O_y_ size, volume and surface area, the average potential energies of the atoms, the lattice energies, force-related descriptors applied on the atoms and the average coordination parameters of the Me_x_O_y_ atoms.

Together with the 15 descriptors from the original dataset, the final dataset used for modelling included a total of 77 descriptors (independent variables), which were correlated with cell viability (dependent variable). The list with all descriptors can be found in Table S1 of the supplementary information file.

### 2.3. NanoPharos Database and Data Management

The complete dataset was cleaned, structured and uploaded into the NanoPharos database (https://db.nanopharos.eu/) developed within the Horizon 2020 (H2020) projects NanoCommons [59] and NanoSolveIT [58]. In short, the dataset was checked for any gaps, enriched with molecular and structural descriptors following the needed atomistic computations. The descriptors were grouped based on their origin (physicochemical, structural, molecular and atomistic) for easier study. NanoPharos was designed under the FAIR (Findable, Accessible, Interoperable, Reusable) data principles to offer users high-quality publicly available ready-for-modelling datasets. NanoPharos is accessible through a Representational State Transfer (REST) application programming interface (API) and is able to interact with external databases (e.g., NanoCommons Knowledgebase and NanoSolveIT Cloud) and modelling tools through programmatic access via the API.

The NanoPharos database is designed to enable further development and use for additional relevant purposes. This is achieved by adding appropriate data structures and more interfaces in a modular fashion. The design of this relational database is performed in a standard fashion to allow its expansion and incorporation of additional datasets of varying composition. NanoPharos database offers the possibility for submitted datasets to be enriched automatically with relevant bibliographic, molecular (e.g., crystal structure, electronegativity) and computational descriptors.

The full enriched dataset used for model development can be accessed through: https://db.nanopharos.eu/Queries/Datasets.zul.

### 2.4. Model Development, Validation, Read Across and Domain of Applicability

The Isalos Analytics Platform, powered with the Enalos+ nodes [71], was used for the development and validation of the produced cytotoxicity QNAR model [72,73,74]. To decrease the risk of low-variance data distracting the modelling algorithm, all double-compatible descriptors, which had low variance and did not significantly contribute to the discrimination power of the model, were removed using a low-variance filter [75]. The removal criterion was set to 0.2, meaning that a descriptor was excluded from the analysis if it contained 20% or more values equal to those of another descriptor. All remaining descriptors were normalised using Z-score normalisation to follow a Gaussian distribution with a mean value of 0.0 and a standard deviation of 1.0 [76]. The dataset was then randomly portioned into training and test sets using a ratio of 70%:30%, respectively. The descriptors with the highest significant contribution were identified using the Correlation-based Feature Selection (CfsSubset) algorithm combined with the BestFirst evaluator (see Section S1 in the Experimental Supplementary Information for a short introduction of the two algorithms) [77,78,79].

The Enalos implementation of the *k*-nearest neighbours (Enalos*k*NN, Enalos Chem/Nanoinformatics tools) methodology was applied to the dataset to produce the cytotoxicity (% cell viability) predictive model. *k*NN is an instance-based (lazy) method that predicts the dependent variable based on the distance of the *k* (*k* = 1, 2, 3, …) nearest neighbours, in the features space R^n^, where n is the total number of descriptors used for the prediction. In our case, the Enalos*k*NN was used in regression mode and cytotoxicity prediction was based on the Euclidian distances (similarity measure) of the target variable from its *k* closest neighbours [78]. In the case of nominal descriptors, the Enalos*k*NN node sets the Euclidian distance to 0 if individual values are the same and 1 otherwise [80].

The *k*NN algorithm can be used according to ECHA’s read across framework [81] for NPs as long as the following criteria are fulfilled:

- Gathering of the required descriptors (physicochemical, molecular and atomistic) for each NP.
- Construction of a data matrix including properties and endpoints.
- Development of an initial grouping hypothesis that correlates an endpoint, to different behaviour and reactivity properties. Assignment of the samples to groups.
- Assessment of the applicability of the approach using computational techniques and data gap filling. If no regular pattern emerged, an alternative grouping hypothesis must be proposed.
- If the grouping hypothesis is robust, but adequate data are not available, additional testing should be considered.
- Justification of the method.

The Euclidean distance can, thus, be used as a metric to identify the dependent’s variable neighbours and predict NP cytotoxicity. By identifying groups of neighbours, it is also possible to divide the entire dataspace into subgroups as per ECHA’s read across framework requirements. The Enalos*k*NN node was built to provide the specific neighbours and Euclidian distances along with the respective predictive results.

The produced model was validated and documented following the OECD’s principles for the validation of predictive models for regulatory purposes [82]. Internal and external validation took place using the goodness-of-fit, robustness and predictivity metrics [83,84]. Statistical evaluation of the model’s performance took place using Trophsa’s tests, i.e., the coefficient of determination between experimental values and model predictions (R^2^), validation through an external test set, leave-many-out cross validation procedure and Quality of Fit and Predictive Ability of a continuous predictive Model [85]. To perform the evaluation, the Enalos Model Acceptability Criteria were used, where the following equations to calculate Tropsha’s tests were implemented:

(1) $$R_{cvtext}^{2}=1-\;\frac{\sum_{i=1}^{ntest}{\left(y_{i}-\tilde{y_{i}}\right)}^{2}}{\sum_{i=1}^{ntest}{\left(y_{i}-{\bar{y}}_{tr}\right)}^{2}}$$

(2) $$k=\frac{\sum_{i=1}^{ntest}y_{i}{\tilde{y}}_{i}}{\sum_{i=1}^{ntest}{\tilde{y}}_{i}{}^{2}}$$

(3) $$R_{o}^{2}=1-\;\frac{\sum_{i=1}^{ntest}{\left({\tilde{y}}_{i}-{\tilde{y}}_{i}^{ro}\right)}^{2}}{\sum_{i=1}^{ntest}{\left({\tilde{y}}_{i}-\bar{\tilde{y}}\right)}^{2}},\;\mathrm{where}\;{\tilde{y}}_{i}^{ro}=ky_{i},\;i\;=\;1,\;2,\;\ldots ,\;\mathrm{ntest},$$

where ntest is the number of NPs in the test set, ${\bar{y}}_{tr}$ is the average cytotoxicity for the training set; $y_{i}$, ${\tilde{y}}_{i}$, *i* = 1, 2, …, ntest are the experimentally measured and the predicted cytotoxicity values for the validation set, respectively and $\bar{\tilde{y}}$ is the average predicted cytotoxicity over all of the predictions ${\tilde{y}}_{i}$, *i* = 1, 2, …, ntest.

Furthermore, according to Tropsha et al. [85], a QSAR model is considered predictive if all of the below conditions are satisfied:

(4) $$R_{cvtext}^{2}>0.5$$

(5) $$R_{pred}^{2}>0.6$$

(6) $$\frac{R_{pred}^{2}-R_{o}^{2}}{R_{pred}^{2}}<0.1$$

(7) 0.85 ≤k ≤1.15.

To ensure that the produced model was not a result of chance correlation and confirm its statistical significance and robustness, we performed Y-randomisation [86]. Ten different datasets were produced following random shuffling of the cytotoxicity predictions and using all original descriptors. The calculations were then repeated several times, and the model acceptability criteria, as described above, were recalculated. For the model to be valid, the recalculated criteria were expected to reduce when compared to the original model.

Finally, to ensure the applicability of the produced model to external datasets and to launch the webservice, the reliability limits, i.e., the domain of applicability (APD), for future predictions was identified. Any predictions made outside the defined limits will be flagged as unreliable [72]. Using the Euclidian distances of all NPs in the training set, the APD can be calculated using:

APD = *<d>* + *Zσ*, (8)

where *<d>* and *σ* are the average and standard deviation of all Euclidian distances in the training set, respectively. *Z* is an empirical cut-off value, which was set to 0.5 [83].

To fully demonstrate that the produced cytotoxicity model meets the OECD criteria, as listed above, we have included in S3 of the ESI a completed QSAR Model Reporting Format (QMRF) template.

## 3. Results and Discussion

The goal of this study is to test whether NP cytotoxicity can be predicted using a combination of physicochemical, molecular and whole NP computational descriptors. The dataset used, by Zhang et al. (2012) [45], contained 24 different Me_x_O_y_ NPs (17 commercial and 7 synthesised in house) along with their physicochemical and structural characterisation (core size, specific surface area, total surface area, hydrodynamic size, ζ-potential, point of zero ζ-potential, metal dissolution and crystal structure) and enriched with a number of molecular descriptors (*E_V_*, *E_C_*, *E_g_*, *χ_cation_*, *χ_oxide_* and *E_ΔH_*). The dataset was enriched further with 62 computational descriptors for each NP, which were derived from chemical composition (9 descriptors), potential energy (9 descriptors), topology (9 descriptors), lattice energy (5 descriptors), size (3 descriptors) and force vectors (27 descriptors). The final dataset included a total of 77 descriptors (independent variables), which were studied in terms of their correlation to % cell viability (dependent variable) of BEAS-2B and RAW 264.7 cell lines exposed to the NPs for 24 h. One of the descriptors used during model development was the assay type (ATP or LDH) used to measure cell viability. The reason for including the assay type was its statistical significance on the produced results, as demonstrated in previous meta-analysis studies [60,63,64,65]. As a result, extra care needs to be taken when dataset combination takes place regarding their interoperability to flag sources of potential variability. This also emphasises the need for sufficient metadata implementation [87] with published datasets to increase their FAIRness score and thus reusability [88].

The produced predictive model was developed following a random division of the dataset into training and test sets (70%:30% respectively). Out of the 77 descriptors used (for a full list see Table S1 in the Supplementary Information) in the dataset, 9 double-compatible (see Table S1 in ESI for list) were removed for having low variance, which risked distracting the used *k*NN algorithm. Following Z-score normalisation of the training set, the retrieved normalisation parameters were used to normalise the test set. The CfsSubset algorithm combined with the BestFirst evaluator [77,78] were then used to identify the descriptors contributing the most to dataset variability (which correlates with model predictivity). From the 61 remaining descriptors 7 were identified as the most significant. These were: NP core size, NP hydrodynamic size, type of assay, NP exposure dose, conduction band energy (*E_c_*), average coordination number of metal atoms in the surface region of the NP (Avg. C.N. Me atoms surface) and average length of surface normal component of force vector of atoms in the surface region of the NP (v⊥ Me atoms surface).

As can be derived from the above, a good balance exists between the physicochemical, assay-related and molecular/atomic descriptors, i.e., 2:2:3, respectively, as drivers of the NP cytotoxicity. More specifically, the physicochemical descriptors are both size related and combine the size of the pristine NP core (as measured with electron microscopy) with the NP’s behaviour within a specific medium (hydrodynamic diameter). NP core size was linked with cytotoxicity in the past [89,90,91,92,93,94] with decreasing size related to higher toxicity. This was usually in conjunction with the assay parameter of exposure dose, since on a constant mass basis there will be much higher numbers of smaller particles relative to larger ones [95]. Studies on BEAS-2B cells [89,90], which included LDH activity, demonstrated that SiO_2_ NPs with a nominal size of 10 nm had higher ability to induce the pro-inflammatory cytokines CXCL8 and IL-6 compared to NPs with a nominal size of 50 nm [89]. Similarly, Li et al. (2016) demonstrated that NPs had higher cytotoxicity and autophagy dysfunction in human bronchial epithelial BEAS-2B cells when compared to an equivalent mass of microscale particles [90], with both end-points varying in a size- and dose-dependent manner. RAW 264.7 cells [91,93] also demonstrated a size- and dose-dependent relation with cytotoxicity, with smaller particles related to higher toxicity. Makama et al. (2018) found that the Ag NP size-dependent toxicity was evident for the production or reactive oxygen species (ROS) [91], while Loan et al. (2018) demonstrated higher toxicity of Au NPs (5 vs. 30 nm nominal sizes) [93].

The fact that exposure dose also plays a significant role in Me_x_O_y_ NP cytotoxicity is expected, since dose–response relationships are at the heart of toxicity evaluation, although in the case of NPs, the relationships are not always linear. High NP concentrations can lead to particle agglomeration which changes the uptake potential and impacts processes such as dissolution. There has also been significant debate as to the most relevant dose metric for NPs, with particle number/cell or particle number/mm^2^ proposed as alternatives to mass [95,96]. Au NPs inactivated the DNA repair system, generating dose-dependent DNA ladder bands on agarose gel electrophoresis [93]. Similar results regarding a range of NPs, including Ag, Al, carbon black, carbon-coated Ag and Au NPs, were also observed by Nishanth et al. (2011) [94].

Similarly, previous studies found correlations between cytotoxicity and the NPs’ hydrodynamic size [97,98,99,100,101]. In most cases, the hydrodynamic size of the NPs is larger than the core size. The hydrodynamic size (*R_H_*) is calculated using the Stokes–Einstein radius equation (Equation (9)) [102]:

(9) $$R_{H}=\frac{k_{B}T}{6\pi \eta D},$$

where *k_B_* is the Boltzmann constant, *T* is the temperature, *η* is the liquid’s viscosity and *D* is an ion’s diffusion coefficient, which is proportional to the ion’s mobility, *μ* (Equation (10)):

(10) $$D=\frac{{\mu k}_{B}T}{q},$$

where *q* is the ion charge and *μ* is correlated (Equation (11)) with *ze*, which is the ionic charge in integer multiples of electron charges and the medium’s frictional coefficient *f* (Equation (12)):

(11) $$\mu =\frac{ze}{f}$$

(12) $$F_{Drag}=fs=\left(6\pi \eta \alpha \right)s,$$

where *F_Drag_* is the drag force applied to a perfect sphere travelling through a viscous liquid of frictional coefficient *f*, *s* is the sphere’s drift speed and *α* is its radius.

Equations (8)–(11) demonstrate the effect of the exposure medium characteristics on the NPs properties and behaviour. In complex media, the ionic charge and diffusion coefficient will attract free radicals and biomolecules leading to the formation of protein corona [97,103,104]. In this way, the NP’s hydrodynamic size and, subsequently, the way in which the NP will interact with biological organisms will change [100]. Dassler et al. showed that decreasing Fe_2_O_3_ NPs hydrodynamic size significantly increased the blood half-life time and biodistribution of these NPs, leading to an alteration of their toxic effects on different organs [98]. This is probably due to the increased potential for NP degradation inside the organism, leading to the release of ions and increased intracellular ROS levels, as shown by Abakumov et al. [99]. Furthermore, increased hydrodynamic size may correspond with NP agglomeration, which can reduce uptake and result in decreased NP toxicity [105].

From the theoretical descriptors, *E_c_* has been correlated with NP toxicity due to its connection to the energy band gap (*E_G_*). Zhang et al. (2012) demonstrated that the overlap of *E_G_* with the cellular redox potential was strongly correlated to the ability of NPs to induce oxidative stress and acute pulmonary inflammation in mice [45]. *E_c_* represents the lowest unoccupied molecular orbital (LUMO) that participates in electron transfers from and to the Me_x_O_y_ surface, while the valence band (*E_V_*) is usually occupied. Thus, if the cellular redox potential is higher than the conduction band edge of the Me_x_O_y_ NP, direct electron transfer from the aqueous electron donor to the conduction band can take place. Alternatively, electrons injected from an aqueous donor could be transferred to the NP and from there to a series of ambient electron acceptors inside the cell until a steady state is reached [45].

**Figure 1:**
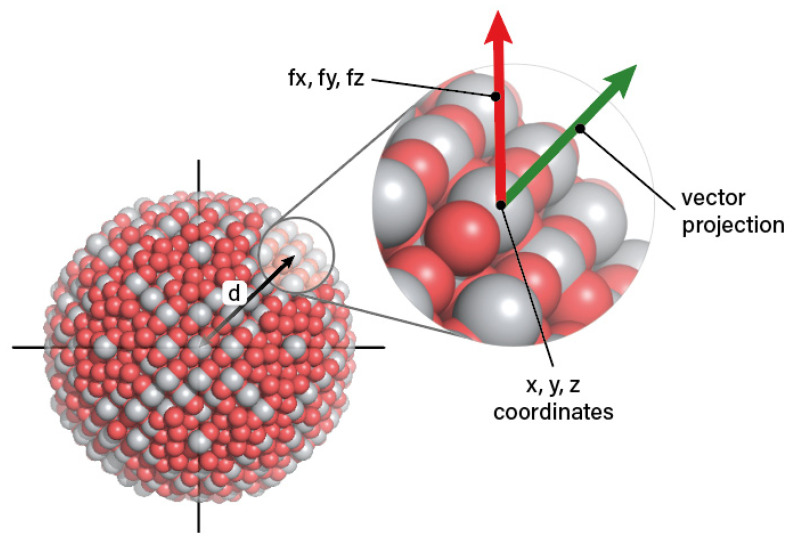
Normal component of the force vector of a single atom (green) in the shell region of a metal oxide (Me_x_O_y_) nanoparticle (NP) (grey spheres: metal atoms and red spheres: oxygen atoms). The average length *V* of the surface normal component of force vector of all atoms in the shell region of the NP is calculated for all atoms using *x*, *y* and *z* coordinates of atom and *f_x_*, *f_y_* and *f_z_* components of the force vector using the formula V = (x*f_x_ + y*f_y_ + z*f_z_)/d, where *x, y* and *z* are atom coordinates relative to the NP centre-of-mass, *f_x_, f_y_* and *f_z_* are the *x*, *y* and *z* components of atoms force vector and d is the distance of the atom from the centre of the NP.

The coordination number of metal atoms in the shell region (Avg. C.N. Me atoms surface) defines the number of all atoms lying within radius *R* of a spherical particle [40]:

(13) $$R=1.2\left(R_{M}+R_{O}\right),$$

where *R_M_* and *R_O_* are the ionic radii of the metal and oxygen ions, respectively. The coordination number of metal atoms is, thus, related to the distance between the metal atoms on the surface of the NP and is directly related to the metal atoms’ stability and dissociation potential. The coordination number corresponds to the chemical bonding (ionic or covalent) in the NP, which is directly correlated to the potential mode of toxicity associated with NP dissolution and ion release. Smaller values of this descriptor indicate that metal atoms are prone to dissociate from the NP surface, releasing ions into the surrounding environment and thus having the potential to cause toxicological effects [40,53]. Similarly, the force vector surface normal component of atoms (metals and oxygens) in the shell region (v⊥ Me atoms surface) describes the strength of bonds between the surface atoms and the NP core, as shown in Figure 1, and can help distinguish between the regular (bulk) NP atoms and those demonstrating the high surface activity properties of NPs [53]. Hence, these properties describe the potential energy (stability and activity) of the atoms on the surface of NPs, with smaller force vector values indicating a more thermodynamically stable surface [53]. As seen in Figure 1, the average length *V* of the surface normal component of the force vector of an atom in the shell region and at distance *d* from the centre of the NP is calculated using the atomic coordinates relative to the centre-of-mass of the NP (*x*, *y* and *z*) and the respective components of the force vector (*f_x_*, *f_y_* and *f_z_*) using Equation (14):

(14) $$V=\frac{xf_{x}+yf_{y}+zf_{z}}{d}.$$

The workflow developed through the Isalos Analytics Platform provided the opportunity to test different predictive algorithms (e.g., J48, random forest and *k*NN) and allowed us to pick *k*NN as the best performing, with *k* = 2 (i.e., three neighbours). In this case, the coefficient of determination ($R_{Pred}^{2}$) of the experimental values versus the model prediction on the test set was *R*^2^ = 0.91. Model validation took place following the OECD’s guidelines and successfully passed Tropsha’s tests [85] (Table 1) demonstrating the robustness and predictivity of the model. As mentioned in the experimental section, the calculation of the test parameters took place using the regression results of the experimentally measured and the predicted values of BEAS-2B and RAW 264.7 cell viability and vice versa. In good agreement, Y-randomisation demonstrated the model’s robustness and validity. Based on the calculated APD value of 2.645, all predictions in our case were classified as reliable (normalised test set range: 0–0.591). These results can act as a guide for potential limitations of the model and provide future users with an indication of their predictions’ reliability.

**Figure 2:**
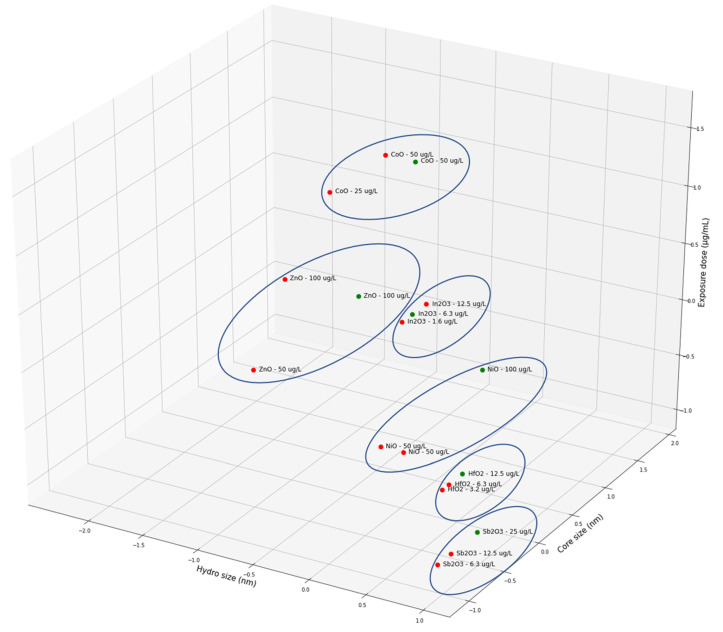
Indicative results for Me_x_O_y_ (CoO, ZnO, In_2_O_3_, NiO, HfO_2_ and Sb_2_O_3_) NPs from the normalised *k*-nearest neighbours (*k*NN) space of the produced cytotoxicity predictive model. The NPs are placed based on their Euclidian distances. Red and green spheres correspond to NPs from the training and test sets, respectively.

The acquired results provided a good picture of the neighbouring space relative to the Euclidian distance between the NP descriptors (specific examples in Figure 2), which were provided by the Enalos*k*NN node. Results demonstrated grouping patterns among NPs with the same core material, indicating the significance of the theoretical descriptors, which are element (and thus NP) specific, and the usage of only three neighbours (*k* = 2) to perform the predictions.

**Figure 3:**
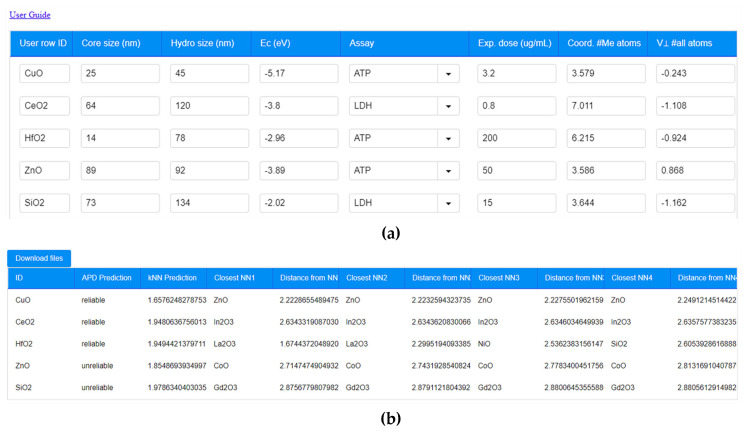
(**a**) Cell viability webservice for the prediction of Me_x_O_y_ NP cytotoxicity. The user inputs the required parameters and the calculation is performed automatically. (**b**) The results provided from the webservice include the prediction, the distances of the closest neighbours and whether the prediction falls within the model’s domain of applicability (APD) (reliable prediction) or not (unreliable prediction).

Taking into account the significance of NP toxicity for hazard and risk assessment and for the safe by design of NPs, the proposed model has been made available through the NanoSolveIT Cloud Platform [106]. The corresponding webservice can be found at: https://cellviability.cloud.nanosolveit.eu/. The service is designed to offer a user-friendly experience (Figure 3a) and requires the input of the 7 parameters identified as significant for prediction of NP cytotoxicity: core size, hydrodynamic size, assay type, exposure dose, conduction band energy, the coordination number of metal atoms (Avg. C.N. Me atoms surface) and the force vector surface normal component of metal atoms (v⊥ Me atoms surface). Indicative values for the theoretical descriptors are offered through the webservice tutorial. Upon submission of the input data—via the graphical user interface (GUI), which requires the specific set of information to be submitted in a specific order, as shown in Figure 3a—calculations are performed automatically. The results (Figure 3b) are provided along with the Euclidian distances of the neighbours and the predictions reliability based on the calculated APD. All results appear on screen and can also be downloaded as a .CSV file.

The produced model is complemented with a REST API to make it available and easy to use programmatically, i.e., to implement into a workflow, e.g., in KNIME nodes. The REST API is used to communicate with the Isalos analytics platform and request the data submission and exchange necessary to run the model. As the model requires a large amount of data to be transferred, the API has been implemented using the POST Request Method. This method includes in the body of the request the user submitted data to be used to make the prediction. These can be either typed into the webservice’s GUI, as shown in Figure 3a, or uploaded using a .csv file (see the webservice tutorial for more information, which is available at https://cellviability.cloud.nanosolveit.eu/2/instructions.zul). Following submission, the service will provide the produced results in JSON format.

To use the Me_x_O_y_ NP cytotoxicity API, the user is required to form a tuple of data (i.e., a collection of data, which is ordered and immutable) in JSON format. Hence, the tuple should contain “User row ID,” “Core size (nm),” “Hydro size (nm),” “Ec (eV),” “Assay,” “Exp. dose (ug/mL),” “Avg. C.N. Me atoms surface” and “v⊥ Me atoms surface,” which is the same order of input as in the GUI (Figure 3a). Assuming that the first NP shown in Figure 3a (CuO) is submitted, the request will translate, in correspondence to the GUI presented in Figure 3a, to:

{

 “id”: “CuO”,

 “coreSize”: 25,

 “hydroSize”: 45,

 “ecEv”: -5.17,

 “assay”: “ATP”,

 “exposureDose”: 3.2,

 “des306”: 3.579,

 “des606”: -0.243,

}

To use the web API, the user can make a request (submit the desired information) using a data transfer software like Client URL (cURL):

curl -d ‘[{“id”:”CuO”,”coreSize”:25,”hydroSize”:45,”ecEv”: -5.17,”assay”: “ATP”,”exposureDose”:3.2,”des306”:3.579,”des606”:-0.243]’ -H ‘Content-Type: application/json’ https://cellviability.cloud.nanosolveit.eu/

Based on the submitted information, the model will use existing settings to normalise the data to meet the model’s needs and compute the closest neighbours, corresponding Euclidian distances and model reliability (APD). The normalised response along with the results, in this case, will be:

[

{“id”:”CuO”,

“assay”:”ATP”,

“apdPrediction”:”reliable”,

“nn1ID”:”ZnO”,

“nn2ID”:”ZnO”,

“nn3ID”:”ZnO”,

“nn4ID”:”ZnO”,

“coreSize”:-0.11951770136760874,

“hydroSize”:-2.642180718449538,

“ecEv”:-1.6267973637452349,

“exposureDose”:-0.6247745075500192,

“des306”:-1.2112713652065334,

“des606”:0.44321784950783655,

“nn1Distance”:2.222865548947517,

“nn2Distance”:2.2232594323735833,

“nn3Distance”:2.227550196215906,

“nn4Distance”:2.2491214514422158,

“knnprediction”:1.6576248278753694}

]

Similarly, the user can use the REST API to submit more than one request (tuples). To do so, the user needs to separate each tuple with commas. Assuming the user would like to submit the first two lines in Figure 3a, the JSON request would be:

curl -d ‘[{“id”:”CuO”,”coreSize”:25,”hydroSize”:45,”ecEv”: -5.17,”assay”: “ATP”,”exposureDose”:3.2,”des306”:3.579,”des606”:-0.243},{“id”:”CeO2”,”coreSize”:64,”hydroSize”:120,”ecEv”: -3.8,”assay”: “LDH”,”exposureDose”:0.8,”des306”:7.011,”des606”:-0.1108}]’ -H ‘Content-Type: application/json’ https://cellviability.cloud.nanosolveit.eu/

and the corresponding results returned would be:

[

{“id”:”CuO”,”assay”:”ATP”,”apdPrediction”:”reliable”,”nn1ID”:”ZnO”,”nn2ID”:”ZnO”,”nn3ID”:”ZnO”,”nn4ID”:”ZnO”,”coreSize”:-0.11951770136760874,”hydroSize”:-2.642180718449538,”ecEv”:-1.6267973637452349,”exposureDose”:-0.6247745075500192,”des306”:-1.2112713652065334,”des606”:0.44321784950783655,”nn1Distance”:2.222865548947517,”nn2Distance”:2.2232594323735833,”nn3Distance”:2.227550196215906,”nn4Distance”:2.2491214514422158,”knnprediction”:1.6576248278753694},

{“id”:”CeO2”,”assay”:”LDH”,”apdPrediction”:”reliable”,”nn1ID”:”CoO”,”nn2ID”:”CoO”,”nn3ID”:”CoO”,”nn4ID”:”CoO”,”coreSize”:1.9605633643433438,”hydroSize”:-1.505175370072615,”ecEv”:-0.3275969616463464,”exposureDose”:-0.6626005904429065,”des306”:1.6555169751247294,”des606”:0.6598914572620428,”nn1Distance”:1.9192283066219589,”nn2Distance”:1.9208125182623592,”nn3Distance”:1.927695680036072,”nn4Distance”:2.0696148079478345,”knnprediction”:1.9327703185014482}

]

## 4. Conclusions

In this study, we presented a robust, validated and easily applicable model for the prediction of the cytotoxicity of Me_x_O_y_ NPs. The model was developed using a dataset containing 15 physicochemical and structural descriptors, enriched with 62 atomic computational descriptors using the Isalos Analytics Platform and the Enalos+ nodes. Out of the 77 total descriptors used as input, 7 were deemed statistically significant. These are two experimental parameters (core and hydrodynamic size of NPs), two assay-related parameters (assay type (LDH or ATP) and exposure dose) and three computational descriptors: the energy of the conduction band (*E_C_*), the coordination number of surface metal atoms (Avg. C.N. Me atoms surface) and the force vector surface normal component of metal atoms (v⊥ Me atoms surface). *E_C_* can be found from libraries of physicochemical descriptors (e.g., https://materialsproject.org/), and Avg. C.N. Me atoms surface and v⊥ Me atoms surface can be calculated using molecular dynamics software (e.g., LAMMPS). The model allows read across based on chemical similarity of specific Me_x_O_y_ and the use of the LDH or ATP assays to predict cytotoxicity. The curated dataset used in this study, including the values of Avg. C.N. Me atoms surface and v⊥ Me atoms surface, is directly accessible from the NanoPharos database and the model developed is also publicly available as a webservice through NanoSolveIT Cloud Platform.

## Figures and Tables

**Table 1:** Model validation criteria and acquired results.

Criterion	Result	Assessment
R^2^ > 0.6	0.91	Pass
R_cvext_ > 0.5	0.904	Pass
$\frac{R^{2}-R_{0}^{2}}{R^{2}}<0.1$	0.022	Pass
$\frac{R^{2}-R_{0}^{\prime 2}}{R^{2}}<0.1$	0.002	Pass
$\left|R^{2}-R_{0}^{\prime 2}\right|<0.3$	0.018	Pass
0.85 < k < 1.15	0.994	Pass
0.85 < k’ < 1.15	1.005	Pass

## Supplementary Materials

The following are available online at https://www.mdpi.com/2079-4991/10/10/2017/s1. Table S1. List of physicochemical, molecular and atomistic (computational) and assay descriptors used for model development.; Figure S1. The workflow picture for the full-particle structural and energetic nanodescriptor simulation. Overview of Correlation based Feature Selection method combined with the BestFirst evaluator applied to the dataset, which identified the 7 most significant descriptors that were used for model development(S1); Documenting the model using the MODA template (S2); QSAR model report following OECD template (S3).
