# Supplementary material for: Predicting Cytotoxicity of Metal Oxide Nanoparticles Using Isalos Analytics Platform
Source: Nanomaterials (Basel). 2020 Oct 13;10(10):2017. doi: 10.3390/nano10102017 (PMC7601995; doi:10.3390/nano10102017)
Supplement: Supplementary file 1 [file nanomaterials-10-02017-s001.pdf]

Table S1. List of physicochemical, molecular and atomistic (computational) and assay descriptors used for model development.

#	Name	
**Physicochemical descriptors**		
1	Core size	CS
2	Specific surface area	SSA
3	Total surface area	TSA
4	Hydrodynamic size	HD
5	ζ-potential	ZP
6	Point of zero ζ-potential	PZZP
7	Crystal structure	CS
**Molecular descriptors**		
8	Energy of the metal oxide valence band	E_v
9	Energy of the metal oxide conduction band	E_C
10	Energy band gap	E_G
11	Metal electronegativity	χ_{cation}
12	*Me_xO_y absolute electronegativity	χ_{oxide}
13	Standard enthalpy of formation	$E_{\Delta H}$
**Atomistic (computational) descriptors**		
14	Log of total No. of atoms in NP	log(No. atoms all)
15	Log of total No. of atoms in core region of NP	log(No. atoms core)
16	Log of total No. of atoms in surface region of NP	log(No. atoms surface)
17	Log of total No. of Me atoms in NP	log(No. Me atoms all)
18	Log of total No. of Me atoms in core region of NP	log(No. Me atoms core)
19	Log of total No. of Me atoms in surface region of NP	log(No. atoms surface)
20	Log of total No. of O atoms in NP	log(No. O atoms all)
21	Log of total No. of O atoms in core region of NP	log(No. atoms core)
22	Log of total No. of O atoms in surface region of NP	log(No. atoms surface)
23	Avg. pot. energy of all atoms in NP in eV	Avg. P.E. atoms all
24	Avg. pot. energy of atoms in core region of NP in eV	Avg. P.E. atoms core
25	Avg. pot. energy of atoms in surface region of NP in eV	Avg. P.E. atoms surface
26	Relative avg. pot. energy of Me atoms in NP in eV	Avg. P.E. Me atoms all
27	Relative avg. pot. energy of Me atoms in core region of NP in eV	Avg. P.E. Me atoms core
28	Relative avg. pot. energy of Me atoms in surface region of NP in eV	Avg. P.E. Me atoms surface
29	Relative avg. pot. energy of O atoms in NP in eV	Avg. P.E. O atoms all
30	Relative avg. pot. energy of O atoms in core region of NP in eV	Avg. P.E. O atoms core

31	Relative avg. pot. energy of O atoms in surface region of NP in e	Avg. P.E. O atoms surface
32	Avg. coordination No. of all atoms in NP	Avg. C.N. atoms all
33	Avg. coordination No. of atoms in core region of NP	Avg. C.N. atoms core
34	Avg. coordination No. of atoms in surface region of NP	Avg. C.N. atoms surface
35	Avg. coordination No. of Me atoms in NP	Avg. C.N. Me atoms all
36	Avg. coordination No. of Me atoms in core region of NP	Avg. C.N. Me atoms core
37	Avg. coordination No. of Me atoms in surface region of N	Avg. C.N. Me atoms surface
38	Avg. coordination No. of O atoms in NP	Avg. C.N. O atoms all
39	Avg. coordination No. of O atoms in core region of NP	Avg. C.N. O atoms core
40	Avg. coordination No. of O atoms in surface region of NP	Avg. C.N. O atoms surface
41	Diameter of the NP in Å	d(NP)
42	Surface area of the NP in Å^2	SA(NP)
43	Volume of the NP in Å^3	V(NP)
44	Lattice energy of NP in eV	LE(NP)
45	Relative lattice energy of NP to bulk material (eV)	LE(NP)-LE(bulk)
46	Lattice energy of NP divided by the diameter of NP	LE(NP)/diam(NP)
47	*Lattice energy of NP per unit surface area	LE(NP)/SA(NP)
48	*Lattice energy of NP per unit volume	LE(NP)/V(NP)
49	Avg. length of force vector for all atoms	*v* atoms all
50	Avg. length of force vector for all atoms in core region	*v* atoms core
51	Avg. length of force vector for all atoms in surface region	*v* atoms surface
52	Avg. length of force vector for all Me atoms	*v* Me atoms all
53	Avg. length of force vector for Me atoms in core region	*v* Me atoms core
54	Avg. length of force vector for Me atoms in surface region	*v* Me atoms surface
55	Avg. length of force vector for all O atoms	*v* O atoms all
56	Avg. length of force vector for O atoms in core region	*v* O atoms core
57	Avg. length of force vector for O atoms in surface region	*v* O atoms surface
58	*Avg. length of force vector surface normal component for all atoms	$v\perp$ atoms all
59	*Avg. length of force vector surface normal component for all atoms in core region	$v\perp$ atoms core
60	*Avg. length of force vector surface normal component for all atoms in surface region	$v\perp$ atoms surface
61	*Avg. length of force vector surface normal component for all Me atoms	$v\perp$ Me atoms all
62	*Avg. length of force vector surface normal component for Me atoms in core region	$v\perp$ Me atoms core

63	Avg. length of force vector surface normal component for Me atoms in surface region	v⊥ Me atoms surface
64	*Avg. length of force vector surface normal component for all O atoms	v⊥ O atoms all
65	Avg. length of force vector surface normal component for O atoms in core region	v⊥ O atoms core
66	Avg. length of force vector surface normal component for O atoms in surface region	v⊥ O atoms surface
67	Avg. length of force vector surface tangent component for all atoms	V∥ atoms all
68	Avg. length of force vector surface tangent component for all atoms in core region	V∥ atoms core
69	Avg. length of force vector surface tangent component for all atoms in surface region	V∥ atoms surface
70	Avg. length of force vector surface tangent component for all Me atoms	V∥ Me atoms all
71	Avg. length of force vector surface tangent component for Me atoms in core region	V∥ Me atoms core
72	Avg. length of force vector surface tangent component for Me atoms in surface region	V∥ Me atoms surface
73	Avg. length of force vector surface tangent component for all O atoms	V∥ O atoms all
74	*Avg. length of force vector surface tangent component for O atoms in core region	V∥ O atoms core
75	Avg. length of force vector surface tangent component for O atoms in surface region	V∥ O atoms surface
	**Assay descriptors**	
76	Type of assay (ATP or LDH)	ATP/LDH
77	Exposure dose (μg/mL)	ExpD

No.: number; NP: nanoparticle; * Corresponds to descriptors with values variability < 20%.

S1. Overview of Correlation based Feature Selection (CfsSubset) method combined with the BestFirst evaluator

The Correlation based Feature Selection (CfsSubset) method is a feature selection method, which identifies the subset of attributes (independent variables) that are highly correlated and most relevant to the target variable (dependent variable) and uncorrelated with each other [1–3]. To achieve this, CfsSubset uses a search algorithm (equation S1) to identify the attribute subsets with the highest correlation (merit) to the dependent variable [3]:

$$G_S = \frac{k\overline{r_{ci}}}{\sqrt{k + k(k-1)\overline{r_{ii'}}}} \tag{1}$$

where G_s is the merit of the specific subset S containing k attributes, r_{ci} is the mean correlation of the attributes with the dependent variable and $r_{ii'}$ is the average intra-attribute correlation.

In this particular work, the CfsSubset method was complemented with the BestFirst evaluator method, which searches the space of the defined subsets using greedy hillclimbing with backtracking facility. Hillclimbing is an iterative process starting with a random subset of attributes and tries to find a better solution by changing the subset contents, while backtracking is the process of discarding a subset attribute when it is determined that it cannot be part of the best possible solution due to lack of uniqueness relative to the other attributes in the subset and thus low predicitivity [2].

S2: Documenting the model using the MODA template

The European Materials Modelling Council (EMMC) developed a template for reporting simulations to enhance their usability and relevance for industry and regulation. NanoSolveIT is committed to building on these templates, and to providing complete documentation for all our model outputs and datasets.

The templates are workflow are available from the EMMC at: https://emmc.info/moda-workflow-templates/. An annotated version which explains what should be included in each of the fields is available here:

https://emmc.info/wp-content/uploads/2016/06/MODA_V2-2016.06.01-annotated.pdf

MODA for *calculation of full-particle structural and energetic NANODESCRIPTORS.*
***Simulated* in the NanoSolveIT project**

OVERVIEW of the SIMULATION			
1	USER CASE	*Structural and energetic nano-descriptors of spherical nanoparticles*	
2	CHAIN OF MODELS	MODEL 1	*Material (atomistic) using molecular dynamics (LAMMPS)*
3	PUBLICATION PEER-REVIEWING THE DATA	*K. Tämm, L. Sikk, J. Burk, R. Rallo, S. Pokhrel, L. Mädler, J. J. Scott-Fordsmand, P. Burk, T. Tamm, Parametrization of nanoparticles: development of full-particle nanodescriptors, Nanoscale, 36, 2016, 16243-16250*<br><br>*J. Burk, L. Sikk, P. Burk, B. B. Manshian, S. J. Soenen, J. J. Scott-Fordsmand, T. Tamm, K. Tämm, Fe-Doped ZnO nanoparticle toxicity: assessment by a new generation of nanodescriptors, Nanoscale, 46, 2018, 21985-21993*	
4	ACCESS CONDITIONS	*The software is released under the GNU General Public License (GPL). LAMMPS - Plimpton, P. "Fast Parallel Algorithms for Short-Range Molecular Dynamics", Journal of Computational Physics Volume 117, Issue 1, 1 March 1995, Pages 1-19.*<br><br>*https://lammps.sandia.gov/* (3 Mar 2020 release)	
5	WORKFLOW AND ITS RATIONALE	*The calculation requires the structure (i.e. coordinates of all atoms) of a nanoparticle and input parameters (Buckingham and Coulomb force field parametrization) to calculate structural and energetic descriptors of the nanoparticle*	

Workflow picture

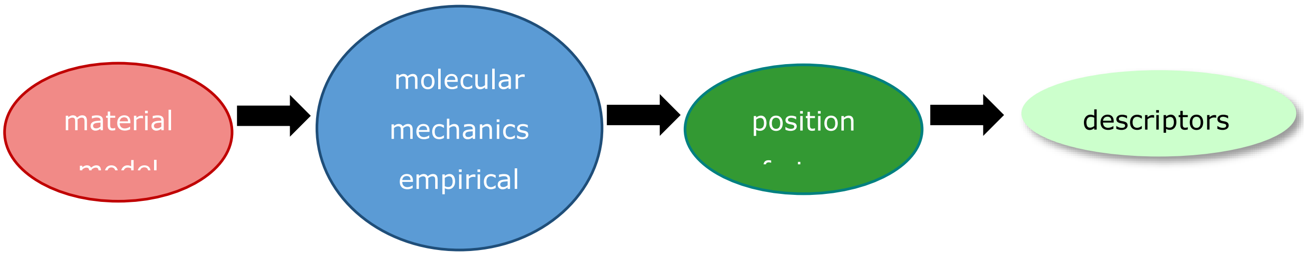

Figure S1. The workflow picture for the full-particle structural and energetic nanodescriptor simulation. At present this model is stand-alone, but in due course all descriptors will be precalculated and integrated into the NanoSolveIT integrated approach to testing and assessment (IATA) at which time the workflow will be extended as these descriptors will serve as inputs to subsequent models.

material

1	Aspect of the User Case/System to be Simulated	
1.1	Aspect of the User Case to be simulated	*Full-particle nano-descriptors calculated using molecular mechanics to evaluate the toxicity of Me_xO_y nanoparticles.*
1.2	Material	*Any material that has the chemical formula Me_xO_y (Me_xO_y where x and y are integers)*
1.3	Geometry	*Spherical system ranging from nanometre to micrometre*
1.4	Time Lapse	*Not applicable*
1.5	Manufacturing process or in-service conditions	*Not relevant*
1.6	Publication on this data	*K. Tämm, L. Sikk, J. Burk, R. Rallo, S. Pokhrel, L. Mädler, J. J. Scott-Fordsmand, P. Burk, T. Tamm, Parametrization of nanoparticles: development of full-particle nanodescriptors, Nanoscale, 36, 2016, 16243-16250.* [10.1039/C6NR04376C](https://doi.org/10.1039/C6NR04376C)

molecular mechanics
empirical force fields

2	Generic Physics Of The Model Equation		
2.0	Model type and name	*Newton's equation-based models – molecular mechanics based on empirical force fields*	
2.1	Model entity	*Atoms*	
2.2	Model Physics / Chemistry equation (PE)	**Equation**	*Newton's equations of motion*
		**Physical quantities**	*Coordinates and mass of each atom, interatomic potentials*
2.3	Materials relations (MR)	**Relation**	*Buckingham and Coulomb potential functions*
		**Physical quantities / descriptors for each MR**	*The Coulomb potential includes the partial charge of each atom and the dielectric constant of the medium in which the material is dispersed*<br>*The Buckingham potential includes atom pair parameters (combination of two elements) and generic parameters such as the cut-off distance*

2.4	SIMULATED INPUT	*Not applicable at present. Will be updated once the model is integrated into the NanoSolveIT IATA.*

position

3	SOLVER AND COMPUTATIONAL TRANSLATION OF THE SPECIFICATIONS		
3.1	NUMERICAL SOLVER	*Energy minimization schemes (e.g. Polak-Ribiere version of the conjugate gradient algorithm) using Particle-Particle Particle-Mesh (PPPM) summation for long-range Coulomb potentials.*	
3.2	SOFTWARE TOOL	*LAMMPS, http://lammps.sandia.gov*	
3.3	TIME STEP	*Not applicable*	
3.4	COMPUTATIONAL REPRESENTATION	PHYSICS EQUATION, MATERIAL RELATIONS, MATERIAL	*The Buckingham and Coulomb potentials are hardcoded as function of atoms type and position.*<br><br>*Atoms are represented are material points.*
3.5	COMPUTATIONAL BOUNDARY CONDITIONS	*periodic boundary conditions expressing infinite domain*	
3.6	ADDITIONAL SOLVER PARAMETERS	• *stopping tolerance for energy (10^{-4} unitless)*<br>• *stopping tolerance for force (10^{-6} in eV/Angstrom)*<br>• *max iterations of minimizer (100)*<br>• *max number of force/energy evaluations (100000)*<br>• *relative error in forces for the PPPM summation (10^{-5} unitless)*	

descriptors

4	POST PROCESSING	
4.1	THE PROCESSED OUTPUT	*The following descriptors are computed:*<br>- *Total number of atoms in the NP*<br>- *Total number of atoms residing in the core of the NP*<br>- *Total number of atoms residing in the surface of the NP*<br>- *For each chemical species, number of atoms of a particular species in the NP*<br>- *For each chemical species, number of atoms of a particular species residing in the core of the NP*<br>- *For each chemical, species, number of atoms of a particular species residing in the surface of the NP*<br>- *Average per-atom potential energy for all atoms in the NP*

		- *Average per-atom potential energy for all atoms residing in the core of the NP*<br>- *Average per-atom potential energy for all atoms residing in the surface of the NP*<br>- *For each chemical species, average per-atom potential energy for all atoms of a particular species in the NP*<br>- *For each chemical species, average per-atom potential energy for all atoms of a particular species residing in the core of the NP*<br>- *For each chemical species, average per-atom potential energy for all atoms of a particular species residing in the surface of the NP*<br>- *Average HEX real part for all atoms in the NP*<br>- *Average HEX real part for all atoms residing in the core of the NP*<br>- *Average HEX real part for all atoms residing in the surface of the NP*<br>- *For each chemical species, average HEX real part for all atoms of a particular species in the NP*<br>- *For each chemical species, average HEX real part for all atoms of a particular species residing in the core of the NP*<br>- *For each chemical species, average HEX real part for all atoms of a particular species residing in the surface of the NP*<br>- *Average HEX imaginary part for all atoms in the NP*<br>- *Average HEX imaginary part for all atoms residing in the core of the NP*<br>- *Average HEX imaginary part for all atoms residing in the surface of the NP*<br>- *For each chemical species, average HEX imaginary part for all atoms of a particular species in the NP*<br>- *For each chemical species, average HEX imaginary part for all atoms of a particular species residing in the core of the NP*<br>- *For each chemical species, average HEX imaginary part for all atoms of a particular species residing in the surface of the NP*<br>- *Average CN for all atoms in the NP*<br>- *Average CN for all atoms residing in the core of the NP*<br>- *Average CN for all atoms residing in the surface of the NP*<br>- *For each chemical species, average CN for all atoms of a particular species in the NP*<br>- *For each chemical species, average CN for all atoms of a particular species residing in the core of the NP*<br>- *For each chemical species, average CN for all atoms of a particular species residing in the surface of the NP*<br>- *Average PTM parameter for all atoms in the NP*<br>- *Average PTM parameter for all atoms residing in the core of the NP*<br>- *Average PTM parameter for all atoms residing in the surface of the NP*<br>- *For each chemical species, average PTM parameter for all atoms of a particular species in the NP*<br>- *For each chemical species, average PTM parameter for all atoms of a particular species residing in the core of the NP*

		- *For each chemical species, average PTM parameter for all atoms of a particular species residing in the surface of the NP*<br>- *Average per-atom force vector length for all atoms in the NP*<br>- *Average per-atom force vector length for all atoms residing in the core of the NP*<br>- *Average per-atom force vector length for all atoms residing in the surface of the NP*<br>- *For each chemical species, average per-atom force vector length for all atoms of a particular species in the NP*<br>- *For each chemical species, average per-atom force vector length for all atoms of a particular species residing in the core of the NP*<br>- *For each chemical species, average per-atom force vector length of a particular species residing in the surface of the NP*<br>- *Average normal component of the per-atom force vector for all atoms in the NP*<br>- *Average normal component of the per-atom force vector for all atoms residing in the core of the NP*<br>- *Average normal component of the per-atom force vector for all atoms residing in the surface of the NP*<br>- *For each chemical species, average normal component of the per-atom force vector of a particular species in the NP*<br>- *For each chemical species, average normal component of the per-atom force vector of a particular species residing in the core of the NP*<br>- *For each chemical species, average normal component of the per-atom force vector of a particular species residing in the surface of the NP*<br>- *Average tangential component of the per-atom force vector for all atoms in the NP*<br>- *Average tangential component of the per-atom force vector for all atoms residing in the core of the NP*<br>- *Average tangential component of the per-atom force vector for all atoms residing in the surface of the NP*<br>- *For each chemical species, average tangential component of the force vector of a particular species in the NP*<br>- *For each chemical species, average tangential component of the per-atom force vector of a particular species residing in the core of the NP*<br>- *For each chemical species, average tangential component of the per-atom force vector of a particular species residing in the surface of the NP*<br>- *NP diameter*<br>- *NP surface area*<br>- *NP volume*<br>- *NP lattice energy*<br>- *NP lattice energy per unit diameter*<br>- *NP lattice energy per unit surface area*<br>- *NP lattice energy per unit volume*<br>- *Relative NP lattice energy to the lattice energy of the bulk material*

		where HEX is hexagonal order parameter, CN is coordination number and PTM is polyhedral template matching.
4.2	**METHODOLOGIES**	*The per-atom potential energy is computed by summing all energetic contributions (pair, bond, angle, etc.) that the atom is part of. Each energy contribution is produced by a small set of atoms (e.g. 2 atoms in a bond or pair-wise interaction, 4 atoms in a dihedral or 3 atoms in a Tersoff 3-body interaction) and the energy is assigned in equal proportions to each atom in the set. E.g. 1/4 of the dihedral energy assigned to each of the 4 atoms and 1/2 of the bond energy assigned to each of the two atoms.*<br><br>*The hexagonal order parameter,* q_n*, of the i*[th] *atom is a complex number defined as*<br>$$q_n = \frac{1}{N}\sum_{j=1}^{N} exp\left(6i\theta(r_{ij})\right)$$<br>*where the sum is over the N = 6 nearest neighbors of the central i*[th] *atom. The angle* θ *is formed by the vector* r_{ij} *and the x axis.* θ *is calculated only using the x and y components, whereas the distance from the central atom is calculated using all three (x, y, and z) components of the bond vector. The complex number* q_n *is restricted to the unit disk of the complex plane i.e.*<br>$$\Re(q_n)^2 + \Im(q_n)^2 \leq 1$$<br>*The coordination number of an atom counts the number of neighbor atoms (irrespective of atom type) that lie within a specified distance from the given atom. The distance is related to the ionic radii of the atoms in the NP; it is equal to 1.2 times the maximum ionic radius of the species present in the NP.*<br><br>*The PTM parameter of atom i is the square-root mean square deviation (RMSD) between its actual position and its position in one of seven perfect lattices. The seven types of lattices are face-centered cubic, hexagonal close-packed, body-centered cubic, icosahedral, simple cubic, diamond cubic, diamond hexagonal, graphene. The identification of the ideal lattice is done employing the polyhedral template matching method.*<br>$$RMSD(u,v) = \min_{s,Q}\sqrt{\frac{1}{N}\sum_{i=1}^{N} \lVert s[u_i - u] - Qu_i \rVert^2}$$<br>*The per-atom force vector length is computed as* $\sqrt{f_x^2 + f_y^2 + f_z^2}$ *where* f *is the per-atom force vector and x, y, z are the three cartesian axes.*<br><br>*The tangential component of the per-atom force vector is computed as* $f_x(r_{i,x} - r_{NP,x}) + f_y(r_{i,y} - r_{NP,y}) + f_z(r_{i,z} - r_{NP,z})$ *where* r_i *and* r_{NP} *are*

		position vectors of the ith atom and the center of mass of the NP. The normal component is computed as the difference between the per-atom force vector and the tangential component.<br><br>*The NP diameter,* D_{NP}*, is computed as twice the maximum distance of an atom from the center of mass of the NP. The NP surface area and volume are calculated as* πD_{NP}^2 *and* $\frac{1}{6}\pi D_{NP}^3$*.*<br><br>*The NP lattice energy is the product of the average per-atom potential energy and the number of atoms in the unit cell. The NP lattice energy relative to the lattice energy of the bulk material is the difference between the average per-atom potential energy of the bulk material and the average per-atom potential energy of the NP multiplied by the number of atoms in the unit cell structure.*<br><br>*The parameters described in the sections above are spatially averaged in three ways:*<br>*(i) Arithmetic average over all atoms belonging to the NP*<br>*(ii) Arithmetic average over all atoms with a distance less than a given radius specified by the user from the center-of-mass of the NP. This region corresponds to the "core" region of the NP*<br>*(iii) Arithmetic average over all atoms which belong to the NP but not to the "core"*<br><br>*There is also averaging with respect to the chemical nature of the various species:*<br>*(i) Arithmetic average over all atoms of the same chemical species*<br>*(ii) Arithmetic average over all atoms irrespective of their chemical species.*
**4.3**	**MARGIN OF ERROR**	*Not applicable*

S3: QSAR model report following OECD template

Cytotoxicity of metal oxide (Me_xO_y) nanoparticles on BEAS-2B and RAW 264.7 cells, developed using the Isalos Analytics Platform, Enalos+ nodes.

In order to demonstrate how the developed models are harmonized with the OECD principles for (Q)SAR model validation for regulatory purposes (OECD, 2004), we summarise here all available information about the development and evaluation of the read-across model in a concise manner. For this purpose, the guidance of the JRC QSAR Model Database is followed, making the necessary alterations for nanoinformatics data (Joint Research Centre, 2017).

Principle 1–A defined endpoint.

Species	Combined human bronchial epithelial (BEAS-2B) and murine myeloid (RAW 264.7) cell lines
Endpoint	Cytotoxic effects of metal oxide (Me_xO_y) nanoparticles (NPs) – 24-hour toxicity to BEAS-2B and RAW 264.7 (measured as cell viability)
Endpoint comments	% cell viability
Endpoint units	% cell viability
Dependent variable	For each effective concentration, the cytotoxicity (% remaining living cells) of Me_xO_ys NPs was calculated.
Experimental protocol	Full experimental description can be found in Zhang et al., ACS Nano. 2012 May 22; 6(5): 4349–4368. doi:10.1021/nn3010087<br><br>Short description: Cell viability was determined by LDH and ATP assays, which were carried out with CytoTox 96® (Promega Corporation, Madison, WI), CellTiter 96® AQueous (Promega Corporation) and ATPliteTM 1step (Perkin Elmer, Boston, MA) assay kits, respectively. Ten thousand cells in 100 µL medium (Dulbecco's Modified Eagle Medium (DMEM) for the RAW cells and bronchial epithelial growth medium (BEGM) for the BAES-2B cells) were plated in each well of a 96 multi-well black plate (Costar, Corning, NY) for overnight growth. The medium was removed and cells treated for 24 hr with 100 µL of a series of Me_xO_y NP suspensions to yield final concentrations of 0.4, 0.8, 1.6, 3.2, 6.3, 12.5, 25, 50, 100 and 200 µg/mL<br><br>24 NP stock solutions (5 mg/mL) were prepared by dispersing the dry particles in deionized water through probe sonication (3 W). The stock solution was used to remove 40 µL aliquots which were mixed with an equal volume of 4% bovine serum albumin (BSA) (Fraction-V, Gemini Bioproducts, USA) and equilibrated for 1 h at room temperature. Cell culture medium (920 µL DMEM) was added to the BSA-coated NP suspensions. The NP suspensions were sonicated (3 W) for 15 seconds prior to conducting cellular studies. For the BEAS-2B exposures, 2 mg/mL of BSA was added to the BEGM for preparation of the series of NP suspensions at different concentrations.
Endpoint data quality and variability	Different NP concentrations were assessed (range 0 – 200 µg/mL)

Principle 2–An unambiguous algorithm.

Type of model	Machine learning, *k*-nearest neighbour (*k*NN) algorithm
Explicit algorithm	Use *k*NN with *k* value equal to 3.
Descriptors in the model	Statistically significant descriptors used for prediction: NP core size, NP hydrodynamic size, assay type, exposure dose, energy of the conduction band (E_C), the coordination number of metal atoms in the NP (Coord. #Me atoms) and

	the force vector surface normal component of atoms ((V⊥ #all atoms).
Descriptor selection	Number and type of descriptors initially screened: 77 descriptors. For full list see Table S1.<br>Method used to select the descriptors: *BestFirst* variable selection along with *CfsSubsetEval* evaluator.
Algorithm and descriptor generation	Experimental measurements (see Zhang et al. (2012) for full details). In summary the measured parameters were % BEAS-2B and RAW 264.7 cell viability (survival) as a function of applied Me_xO_y NP dose at 24 hours. The *k*NN algorithm was used for model generation.
Software name and version for descriptor generation	LAMMPS (https://lammps.sandia.gov/)<br>See ESI Appendix 1 for attached MODA template for atomistic descriptors
NPs/Descriptors ratio	49.29 (345:7, number of data rows divided by the number of significant descriptors in the training set)

Principle 3–A defined domain of applicability.

Description of the applicability domain of the model	The applicability domain (AD) is defined by fixed boundaries (threshold). The threshold is calculated by considering Euclidean distances between all training set NPs.
Method used to assess the applicability domain	Euclidean distance method among all training and test NPs. The distance of each test NP or each external NP to each nearest neighbour of the training NPs set is compared to a predefined AD threshold; if this distance is lower than the threshold then its endpoint prediction can be considered reliable.
Software name and version for applicability domain assessment	Enalos+ nodes version 1.0<br>(see Afantitis et al. (2020), **DOI** : 10.2174/0929867327666200727114410)
Limits of applicability	APD threshold: 2.645.<br>Calculated using the average and standard deviation of all Euclidian distances in the training set. Predictions outside this threshold are considered unreliable.

Principle 4–Appropriate measures of goodness-of-fit, robustness and predictivity.

Availability of the training set	Data available via NanoPharos at https://db.nanopharos.eu/<br>Dataset name: Papadiamantis et al. (2020), Predicting cytotoxicity of metal oxide nanoparticles using Isalos Analytics platform

	Information: Dataset retrieved from S2Nano database (data produced by Zhang et al. (2012, doi:10.1021/nn3010087).<br>Atomistic descriptors produced as per Tamm et al. (2016), doi:10.1039/C6NR04376C.
Available information for the training set	24 tested Me_xO_y NPs: Al_2O_3, CuO, CeO_2, Co_3O_4, CoO, Cr_2O_3, Fe_2O_3, Fe_3O_4, Gd_2O_3, HfO_2, In_2O_3, La_2O_3, Mn_2O_3, NiO, Ni_2O_3, Sb_2O_3, SiO_2, SnO_2, TiO_2, WO_3, Y_2O_3, Yb_2O_3, ZnO, ZrO_2<br>Analytical information on the experimental process can be found in Zhang et al., ACS Nano. 2012 May 22; 6(5): 4349–4368. doi:10.1021/nn3010087
Data for each descriptor variable for the training set	Yes
Data for the dependent variable (response) for the training set	Yes
Other information about the training set	Total of 345 data points for the dependent variable (% cell availability) from 24 NPs: 37 toxic, 308 non-toxic.
Pre-processing of data before modelling	Gaussian normalization of descriptors
Statistics for goodness-of-fit	Tropsha's tests, i.e. the coefficient of determination between experimental values and model predictions (R^2), validation through an external test set, leave-many-out cross validation procedure and Quality of Fit and Predictive Ability of a continuous QSAR Model, as per Tropsha, A., Best Practices for QSAR Model Development, Validation, and Exploitation. Molecular Informatics 2010, 29, (6-7), 476-488 https://doi.org/10.1002/minf.201000061
$R^2 > 0.6$	0.91
$R_{cvext} > 0.5$; Result: 0.904	0.904
$(R^2-R_0^2)/R^2 < 0.1$	0.022
$(R^2-R_0'^2)/R^2 < 0.1$	0.002
$\|R^2-R_0'^2\| <0.3$	0.018
$0.85 < k < 1.15$	0.994
$0.85 < k' < 1.15$	1.005
Robustness – Statistics obtained by Y-scrambling	Accuracy in test = 0.406-0.694 (10 iterations)
Robustness – Statistics obtained by bootstrap	Yes, see above
Robustness – Statistics obtained by other methods	Yes, see above
Availability of the external validation set	Yes

Available information for the external validation set	Yes
Data for each descriptor variable for the external validation set	Yes
Data for the dependent variable for the external validation set	Yes
Other information about the external validation set	Total 149 data points from 24 Me_xO_y NPs.<br>14 toxic and 135 non-toxic NPs
Experimental design of test set	Partition of the initial dataset using random, stratified sampling (70:30 training : test sets)
Predictivity – Statistics obtained by external validation	$R^2 > 0.6$; Result: 0.91<br>$R_{cvext} > 0.5$; Result: 0.904<br>$(R^2-R_0^2)/R^2 < 0.1$; Result: 0.022<br>$(R^2-R_0'^2)/R^2 < 0.1$; Result: 0.002<br>$\lvert R^2-R_0'^2 \rvert <0.3$; Result: 0.018<br>$0.85 < k < 1.15$; Result: 0.994<br>$0.85 < k' < 1.15$; Result: 1.005
Predictivity – Assessment of the external validation set	The external validation set is the 30% of the initial dataset and all predictions for the validation set fall within the domain of applicability
Comments on the external validation of the model	N/A

Principle 5–A mechanistic interpretation.

Mechanistic basis of the model	Exposure dose, NP size and surface characteristics related to the specific metal constituting the NP play a significant role in toxicity of Me_xO_y NPs to BEAS-2B and RAW 264.7 cell toxicities.<br>The cells have different functionalities (epithelial barrier versus phagocytotic, respectively) and as such are expected to internalise NPs to different extents.<br>Care must be taken when predicting results, as the assay type used for experimental data production plays a significant role.
A priori or a posteriori mechanistic interpretation	Me_xO_y NP toxicity can be predicted using a combination of physicochemical, assay-related and atomistic descriptors. Higher doses of NPs lead to higher toxicity. Furthermore, the stability of the NP surface plays a significant role, as the higher the force vector of surface metal atoms the higher the potential for ion leaching and NP dissociation that can lead to toxicity from the release metal cations.
Other information about the mechanistic interpretation	No other information available.

References

1. Hall, M.; Frank, E.; Holmes, G.; Pfahringer, B.; Reutemann, P.; Witten, I.H. The WEKA data mining software: an update. *SIGKDD Explor. Newsl.* **2009**, *11*, 10–18, doi:10.1145/1656274.1656278.
2. Witten, I.H.; Frank, E.; Hall, M.A.; Pal, C.J. *Data Mining: Practical machine learning tools and techniques*; Morgan Kaufmann: Burlington, MA, USA, 2016.
3. Hall, M.A. Correlation-based feature selection for machine learning. Ph.D. Thesis, The University of Waikato, Hamilton, New Zealand, April 1999.
